# Synthesis, *In-Vitro* Antibacterial, Antifungal, and Molecular Modeling of Potent Anti-Microbial Agents with a Combined Pyrazole and Thiophene Pharmacophore

**DOI:** 10.3390/molecules20058712

**Published:** 2015-05-14

**Authors:** Yahia Nasser Mabkhot, Nahed Ahmed Kaal, Seham Alterary, Salim S. Al-Showiman, Assem Barakat, Hazem A. Ghabbour, Wolfgang Frey

**Affiliations:** 1 Department of Chemistry, College of Science, King Saud University, P.O. Box 2455, Riyadh 11451, Saudi Arabia; E-Mails: nahed.a.k@hotmail.com (N.A.K.); salterary@ksu.edu.sa (S.A.); showiman@ksu.edu.sa (S.S.A.-S.); 2 Department of Chemistry, Faculty of Science, Alexandria University, P.O. Box 426, Ibrahimia-21321 Alexandria, Egypt; 3 Department of Pharmaceutical Chemistry, College of Pharmacy, King Saud University, P.O. Box 2457, Riyadh 11451, Saudi Arabia; E-Mail: ghabbourh@yahoo.com; 4 Institut für Organische Chemie, Universitat Stuttgart, Pfaffenwaldring 55, Stuttgart 70569, Germany; E-Mail: wolfgang.frey@oc.uni-stuttgart.de

**Keywords:** thiophene, enaminone, [2+3] cycloaddition, anti-microbial activity, molecular modeling

## Abstract

Ethyl 5-acetyl-4-methyl-2-(phenylamino)thiophene-3-carboxylate (**2**) and there derivatives **3a**–**c**, **4**, **6a**–**c** and **9a**–**f** were synthesized. The structure of compound **2** was deduced by ^1^H-NMR, ^13^C-NMR, FT-IR, MS, microanalysis, and single-crystal X-ray crystallography. The compound crystallized in the monoclinic system, with space group *P*2_1_/*c* and cell coordinates a = 8.5752(16) Å, b = 21.046(4) Å, c = 8.2941(12) Å, β = 101.131(6)°, V = 1468.7(4) Å^3^, and Z = 4. Compounds **2**, **3a**–**c**, **4**, **5a**–**c** and **9a**–**f** were subjected into *in vitro* antimicrobial activity tests. Compounds **3a** and **3c** were more potent than standard drug amphotericin B, showing MIC values of 23.8 ± 0.42 and 24.3 ± 0.68, respectively, against *Aspergillus fumigatus* while the standard drug MIC was 23.7 ± 0.1. Compound **3c** was also more potent (MIC 24.8 ± 0.64) than the standard drug amphotericin B (MIC 19.7 ± 0.2) against *Syncephalastrum racemosum*. Compounds **4** and **9f** also showed promising anti-microbial activity. Molecular modeling was performed for the most active compounds.

## 1. Introduction

Heterocyclic compounds possessing the thiophene core have attracted tremendous interest in the field of medicinal chemistry due to their diverse and wide range of biological properties, including analgesic [1], antidepressant [2], anti-inflammatory [3], antimicrobial [4] and anticonvulsant activities [5,6,7,8]. The thiophene moiety is an integral part of the structure of different antiepileptic drugs (AEDs), for example etizolam, brotizolam, and tiagabine. It is worthy to mention that it has been established that the higher activity of sodium phethenylate has been attributed to the fact the structure contains a thiophene ring. Consequently, the synthesis of novel thiophene analogues has gained much importance in medicinal chemistry due to their potential as labile pro-drugs [7,8,9].

Enaminones are versatile precursors that have a lot of synthetic applications in organic chemistry. Enaminones are key synthons for the synthesis of a wide variety of naturally occurring alkaloids [10,11], and nitrogen-containing heterocycles [12,13,14,15]. They have also been employed as important intermediates for the synthesis of pharmaceutical drugs with antiviral, larvicidal [16] and anticonvulsant properties [17,18,19]. Due to their rich applications, many efficient approaches to these compounds have been developed. In continuation of our research program [20,21,22,23,24] studying the synthesis of novel heterocyclic compound which may be biologically active, herein, we report the synthesis of some novel heterocyclic compounds incorporating a combination of thiophene and pyrazole pharmacophores. The structure of the key intermediate 5-acetyl-4-methyl-2-(phenylamino)-thiophene-3-carboxylate (**2**) was unambiguously deduced by the single-crystal X-ray diffraction technique. New series (**3a**–**c**, **4**, **6a**–**c** and **9a**–**f**) from the key intermediate **2** were synthesized. The antimicrobial activities of the synthesized compounds were also examined and the molecular modeling of the most active products is discussed.

## 2. Results and Discussion

### 2.1. Synthesis of Compounds **2**, **3a**–**c**, **4**, **6a**–**c** and **9a**–**f**

**Scheme 1:**
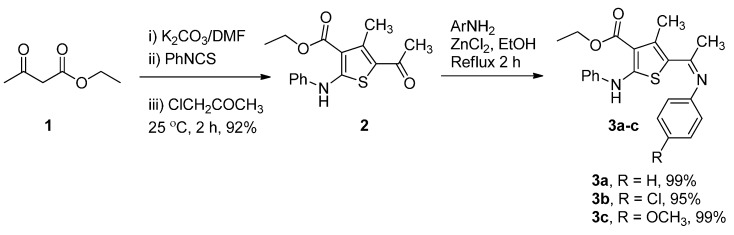
Synthesis of ethyl 5-acetyl-4-methyl-2-(phenylamino)thiophene-3-carboxylate (**2**) and there derivatives **3a**–**c**.

Ethyl 5-acetyl-4-methyl-2-(phenylamino)thiophene-3-carboxylate (**2**) was synthesized in 92% yield as shown in Scheme 1. Reaction of ethyl acetoacetate with phenyl isothiocyanate in the presence of K_2_CO_3_ under reflux in DMF, followed by addition of chloroacetone furnished the product **2**. Compound **2** later reacted with aromatic amines using a catalytic amount of ZnCl_2_ in refluxing EtOH for 2 h to afford regioselectively the Schiff’s bases **3a**–**c** in excellent yields of up to 99%. The structure of **2** was deduced by combined use of IR, ^1^H-NMR, ^13^C-NMR, and mass spectral data. In addition, the assigned structure of **2** was unambiguously established via a single-crystal X-ray diffraction study.

**Scheme 2:**
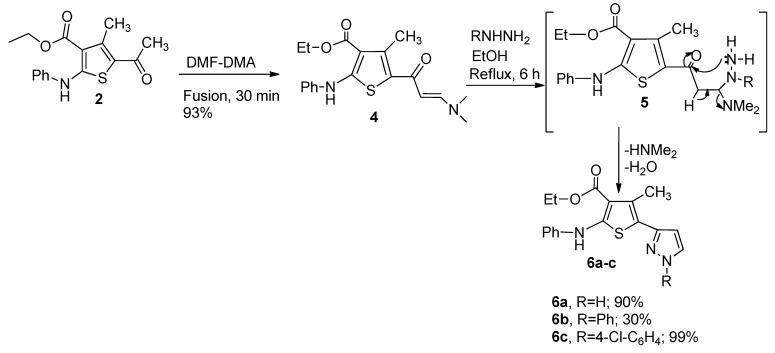
Synthesis of **4** and **6a**–**c**.

**Scheme 3:**
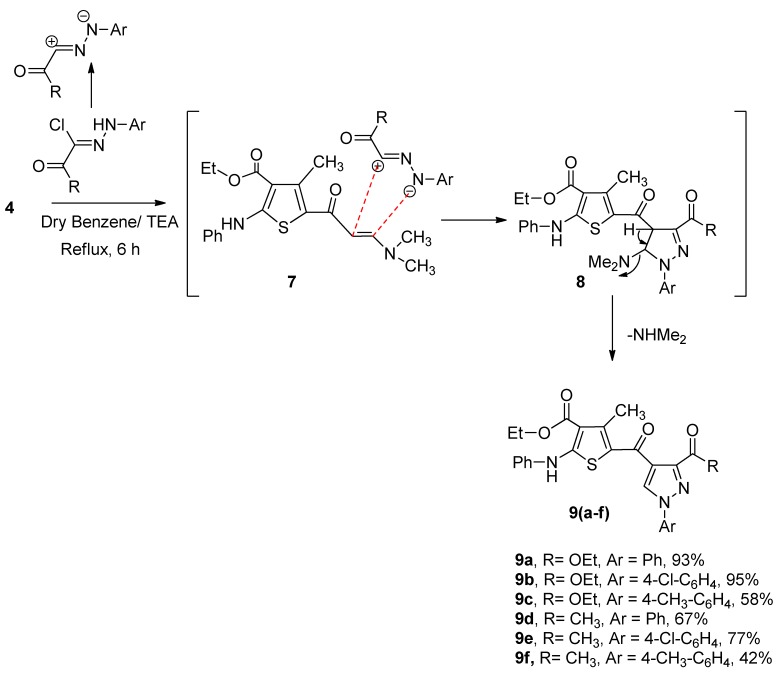
Synthesis of **9a**–**f**.

Next, Condensation of **2** with dimethylformamide dimethyl acetal (DMF-DMA) furnished enaminone **4** in excellent yield 93% (Scheme 2). Reaction of enaminone **4** with an *N*-nucleophile such as hydrazine hydarate, phenyl hydrazine and *p*-chlorophenyl hydrazine in EtOH under reflux for 4–6 h in the presence of a catalytic amount of ZnCl_2_ as a Lewis acid afforded **6a**–**c**, respectively (Scheme 3). The formation of compounds **6a**–**c** would involve an initial addition of the amino group in the hydrazine to the activated double bond in the enaminone derivative **4**, followed by deamination to an intermediate which then undergoes cyclization and aromatization via loss of water affording the final isolable pyrazole derivatives **6a**–**c**.

The utility of enaminone **4** in the synthesis of annulated heterocycles was further explored via its reaction with (*Z*)-ethyl 2-chloro-2-(2-phenylhydrazono)acetate derivatives to afford **9a**–**f** in very good yield (Scheme 3). Spectral data (IR, NMR, MS) and elemental analysis were consistent with the proposed structures of isolated products **9a**–**f**. It is assumed that these products were formed via a [2+3] cycloaddition reaction and initial formation of a nonisolable pyrazole derivative **8**, followed by elimination of NHMe_2_ to give the desired products **9a**–**f**.

### 2.2. X-ray Crystal Structure of Compound **2**

Slow evaporation of an ethanol solution of pure compound **2** afforded colorless crystals. A single crystal of approximate dimensions 0.33 × 0.17 × 0.14 mm was selected for the X-ray diffraction technique Data were collected on a Bruker Kappa APEXII Duo diffractometer equipped with a CCD detector and graphite monochromatic Mo Kα radiation (λ = 0.71073 Å) at 100 K. Cell refinement and data reduction were performed by Bruker SAINT. SHELXS-97 [25,26] was used to solve the structure (Table 1, Table 2 and Table 3). 

**Figure 1:**
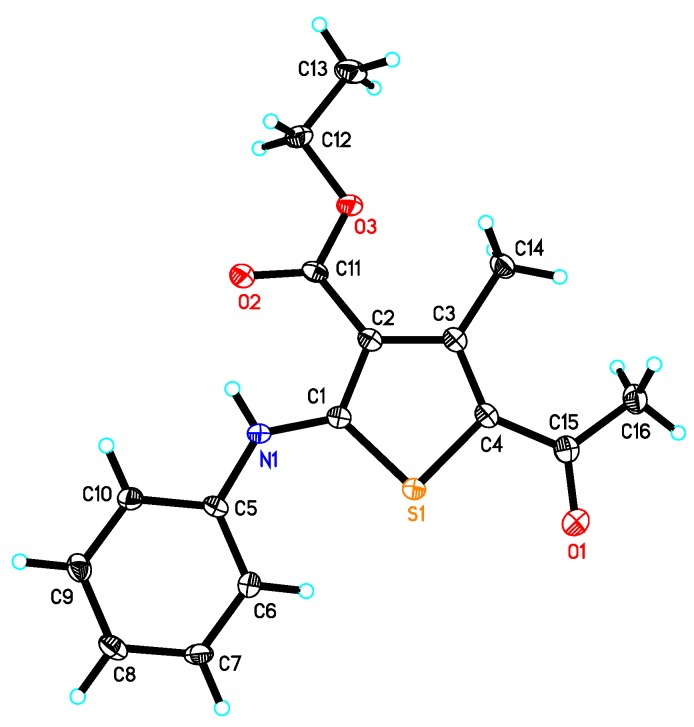
The ORTEP diagram of the final X-ray model of compound **2** with displacement ellipsoids drawn at 50% probability level. H-atoms were placed and not included in refinement, except H1 attached on N1.

The crystal structure of compound **2** is composed of a planar thiophene ring (S1-C2-C3-C4-C5) with phenylamino (N1/C5-C10), ethyl carboxlyate (C11/O2/O3/C12-C13), methyl (C14), and acetyl (O1/C15-C16) substituents attached to the C1, C2, C3 and C4 atoms of the thiophene ring, respectively (Figure 1). 

**Table 1:** The crystal and experimental data of compound **2**.

*Crystal Data*	
C_16_H_17_NO_3_S	*V* = 1468.7 (4) Å^3^
*M_r_* = 303.37	*Z* = 4
Monoclinic, *P*2_1_/*c*	Mo *K*α radiation
*a* = 8.5752(16) Å	µ = 0.23 mm^−1^
*b* = 21.046(4) Å	*T* = 100 K
*c* = 8.2941(12) Å	0.33 × 0.17 × 0.14 mm
β = 101.131(6)°	
*Data Collection*	
Bruker Kappa APEXII Duo diffractometer	2854 reflections with *I* > 2σ(*I*)
Absorption correction: multi-scan Blessing, 1995	*R*_int_ = 0.044
*T*_min_ = 0.684, *T*_max_ = 0.746	θ_max_ = 28.3°
14240 measured reflections	Standard reflections: 0
3620 independent reflections	
*Refinement*	
*R*[*F*^2^ > 2σ(*F*^2^)] = 0.037	0 restraints
*wR*(*F*^2^) = 0.090	H atoms treated by a mixture of independent and constrained refinement
*S* = 1.02	Δρ_max_ = 0.37 e Å^−3^
3620 reflections	Δρ_min_ = −0.24 e Å^−3^
197 parameters	

**Table 2:** Selected geometric parameters (Å, °).

S1—C1	1.7280(14)	C7—H7	0.9500
S1—C4	1.7510(15)	C8—C9	1.390(2)
N1—C1	1.3571(18)	C8—H8	0.9500
N1—C5	1.4075(18)	C9—C10	1.383(2)
N1—H1	0.860(19)	C9—H9	0.9500
O1—C15	1.2335(18)	C10—H10	0.9500
C1—C2	1.413(2)	C12—C13	1.506(2)
O2—C11	1.2294(17)	C12—H12A	0.9900

**Table 3:** Hydrogen bonding data for compound **2**.

D	H	A	D-H	H...A	D...A	D-H…A
N1	H1	O2	0.86(2)	1.91(2)	2.653(2)	143(2)
C6	H6	S1	0.9500	2.46	3.158(2)	130.00
C14	H14B	O3	0.9800	2.4200	2.793(2)	102.00

All hydrogen bonds act intramolecularly for stabilizing the flat geometry of the molecule.

**Figure 2:**
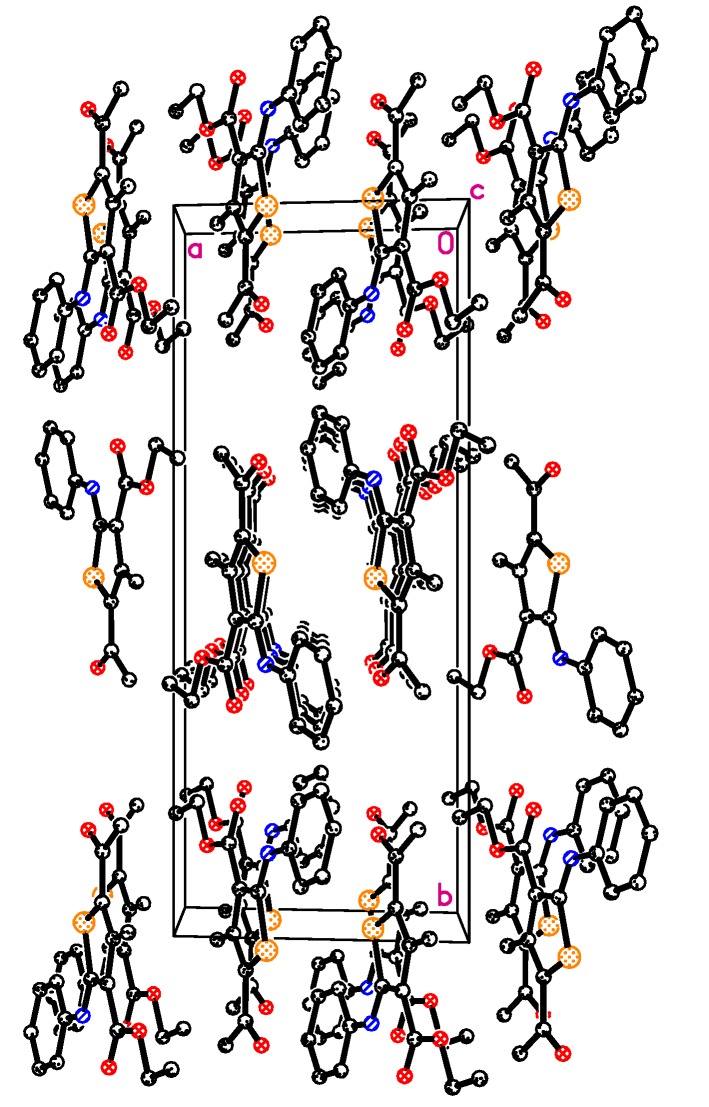
The packing diagram of compound **2** in the crystal lattice. Hydrogen atoms not involved in intermolecular hydrogen bonding are omitted for clarity.

The molecular packing of the compound as observed in Figure 2. All the crystallographic data of the crystal structure **2** (CCDC No. 1042688) are available and can be obtained free of charge from the Cambridge Crystallographic Data Centre via http://www.ccdc.cam.ac.uk/data_request/cif.

### 2.3. Antimicrobial Activity

To investigate the biological activity of the newly prepared compounds, the cup-plate agar diffusion method was adopted by using sterile filter paper discs (6 mm in diameter). Tested compounds were dissolved in DMSO and loaded on the discs at concentrations of 5 mg/mL. The discs were then placed in Petri dishes and were charged with different Gram-positive and Gram negative bacterial strains *Pseudomoas aeruginosa* and *Escherichia coli* for Gram-negative bacteria and *Staphylococcus pneumonia* and *Bacillis subtilis* for Gram-positive, and *Aspergillus fumigates*, *Syncephalastrum racemosum, Geotricum candidum* and *Candida albicans* for fungi. Results of the biological activity are displayed in Table 4; results are expressed as mm inhibition.

**Table 4:** Antibacterial and antifungal activity of synthesized compound (zone of inhibition in diameter in mm).

Compd.			Fungi ^[a]^		Gram (+) Bacteria ^[b]^		Gram (−) Bacteria ^[c]^	
	*(A)*	*(B)*	*(C) *	*(D)*	*(E)*	*(F)*	*(G)*	*(H)*
**2**	13.8 ± 0.42	14.1 ± 0.35	13.2 ± 0.34	10.8 ± 0.22	13.7 ± 0.31	10.5 ± 0.32	11.7 ± 0.41	12.5 ± 0.48
**3a**	23.8± 0.42	13.5 ± 0.29	16.7 ± 0.42	18.1 ± 0.41	20.8 ± 0.54	22.3 ± 0.64	15.3 ± 0.47	18.1 ± 0.62
**3b**	15.9± 0.52	14.5 ± 0.34	16.4 ± 0.35	12.7 ± 0.37	12.2 ± 0.33	13.9 ± 0.52	11.8 ± 0.42	14.7 ± 0.50
**3c**	24.3± 0.68	24.5 ± 0.64	25.8 ± 0.58	14.3 ± 0.58	16.3 ± 0.52	19.6 ± 0.58	12.5 ± 0.39	14.8 ± 0.47
**4**	23.7 ± 0.1	19.7 ± 0.2	28.7 ± 0.2	25.4 ± 0.1	23.8 ± 0.2	32.4 ± 0.3	17.3 ± 0.1	19.9 ± 0.3
**6a**	13.9 ± 0.42	11.8 ± 0.31	13.7 ± 0.34	14.0 ± 0.29	16.9 ± 0.42	17.6 ± 0.31	12.9 ± 0.28	14.7 ± 0.4
**6b**	20.6 ± 0.5	16.7 ± 0.33	22.4 ± 0.36	17.6 ± 0.58	18.3 ± 0.25	22.6 ± 0.44	19.3 ± 0.52	17.8 ± 0.44
**6c**	16.8 ± 0.39	13.4 ± 0.58	19.6 ± 0.19	15.9 ± 0.44	16.7 ± 0.36	19.2 ± 0.27	13.3 ± 0.36	13.6 ± 0.36
**9a**	22.3 ± 0.2	16.5 ± 0.25	25.8 ± 0.58	12.3 ± 0.35	19.5 ± 0.44	29.8 ± 0.58	12.3 ± 0.25	17.6 ± 0.19
**9b**	20.6 ± 0.35	14.8 ± 0.34	21.5 ± 0.62	10.9 ± 0.18	17.8 ± 0.58	20.1 ± 0.39	10.9 ± 0.31	15.3 ± 0.32
**9c**	21.7 ± 0.5	18.1 ± 0.32	20.7 ± 0.34	12.6 ± 0.37	18.7 ± 0.62	24.9 ± 0.46	13.8 ± 0.43	17.1 ± 0.52
**9d**	17.8 ± 0.57	14.6 ± 0.64	18.0 ± 0.72	15.4 ± 0.36	16.9 ± 0.58	18.2 ± 0.44	11.8 ± 0.48	14.2 ± 0.42
**9e**	19.1 ± 0.58	16.7 ± 0.48	14.9 ± 0.63	12.7 ± 0.44	16.8 ± 0.62	17.9 ± 0.48	18.1 ± 0.58	17.4 ± 0.47
**9f**	23.7 ± 0.1	19.7 ± 0.2	28.7 ± 0.2	25.4 ± 0.1	23.8 ± 0.2	32.4 ± 0.3	12.9 ± 0.43	12.7 ± 0.56
**SD-1** ^[d]^	23.7 ± 0.1	19.7 ± 0.2	28.7 ± 0.2	25.4 ± 0.1	-	-	-	-
**SD-2** ^[e]^	-	-	-	-	23.8 ± 0.2	32.4 ± 0.3	-	-
**SD-3** ^[f]^	-	-	-	-	-	-	17.3 ± 0.1	19.9 ± 0.3

^[a]^ *(A)*: *Aspergillus fumigatus*, *(B)*: *Syncephalastrum racemosum*, *(C)*: *Geotricum candidum*, *(D)*: *Candida albicans*; ^[b]^ *(E): Staphylococcus aureus*, *(F): Bacilils subtilis*; ^[c]^ *(G): Pseudomonas aeruginosa*, *(H)*: *Escherichia coli*; ^[d]^ **SD-1**: Amphotericin B for fungi (25 µg/mL); ^[e]^ **SD-2**: Ampicillin for Gram (+) Bacteria (25 µg/mL); ^[f]^ **SD-3**: Gentamicin for Gram (−) Bacteria (25 µg/mL).

The results shown in Table 1 reveal that compounds **2**, **3a**–**c**, **4**, **6a**–**c** and **9a**–**f** exhibit moderate to high activity against both fungi and Gram-positive bacteria. On the other hand, compounds **3a**, **3c** and **9f** were the most active against the tested fungi. Results also show that compounds **3a** and **3c** with MIC values of 23.8 ± 0.42 and 24.3 ± 0.68, respectively, were more potent than the standard drug amphotericin B (MIC 23.7 ± 0.1) against *Aspergillus fumigatus*. On the other hand, **3c** (MIC 24.8 ± 0.64) was also more potent than the standard drug (amphotericin B, MIC 19.7 ± 0.2) against *Syncephalastrum racemosum*. Compound **6b** showed potent activity against *Pseudomonas aeruginosa* with a MIC of 19.3 ± 0.52 while the standard drug gentamicin showed 17.3 ± 0.1. Compounds **4** and **9f** have shown the most promising antifungal as well as antibacterial activity, with MICs of 23.7 ± 0.1, 19.7 ± 0.2, 28.7 ± 0.2, 25.4 ± 0.1, 23.8 ± 0.2 and 32.4 ± 0.3 against *Aspergillus fumigatus*, *Syncephalastrum racemosum*, *Geotricum candidum*, *Candida albicans*, *Staphylococcus aureus*, *and Bacilus subtilis*, respectively. Compound **4** also showed potent activity towards *Escherichia coli* (MIC 19.9 ± 0.3) while the standard drug gentamicin showed a MIC of 19.9 ± 0.3.

### 2.4. Molecular Modeling

To understand the mechanism of the antimicrobial and antifungal activities of the compounds synthesized, molecular modelling and docking studies were performed on the X-ray crystal structure of the *E. coli* 24 kDa domain in complex with clorobiocin (PDB code: 1KZN; resolution 2.30 Å) and cytochrome P450 14α-sterol demethylase from *Mycobacterium tuberculosis* (*Mycobacterium* P450 DM) and co-crystalline fluconazole (PDB code: 1EA1) using the Molegro Virtual Docker (MVD 2013.6.0.0 [win32]) program. In the *E. coli* 24 kDa domain clorobiocin (reference compound) was found to have hydrogen bonding interactions with Asp73 (1.911 Å), Thr165 (2.109 Å), Asn46 (2.034 Å) and Arg136 (2.071 Å) with a MolDock score of −175.0. The fourteen tested compounds revealed MolDock scores between −129.8 to −169.8 (Table 5). 

**Table 5:** MolDock scores for the reference and tested compounds.

Ligand	*E. Coli* 24 kda Domain	Cytochrome p450 14α-Sterol Demethylase
	MolDock Score	MolDock Score
2	−129.864	−146.394
4	−140.52	−144.834
3a	−140.548	−157.378
3b	−149.314	−147.109
3c	−140.293	−139.61
6a	−141.739	−153.137
6b	−146.186	−167.821
6c	−144.798	−175.468
9a	−148.469	−215.797
9b	−167.672	−221.17
9c	−160.583	−213.93
9d	−165.572	−190.91
9e	−168.035	−204.502
9f	−169.884	−167.873
Reference	−175.052	−136.776

**Figure 3:**
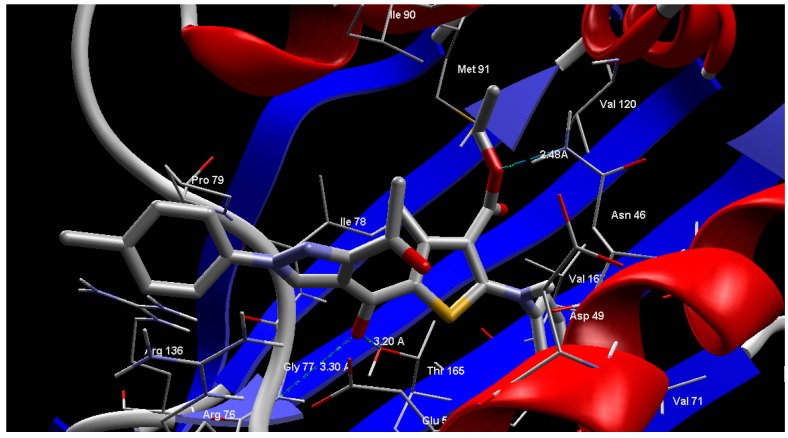
Interaction of compound **9f** with the active site of the *E. coli* 24 kDa domain.

**Figure 4:**
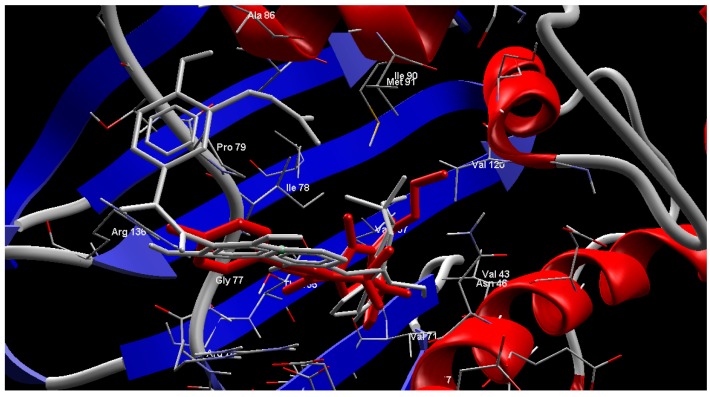
Superimpose of the co-crystallized clorobiocin (**Gray**) and compound **9f** (**Red**) in the active site of the *E. coli* 24 kDa domain.

Compound **9f** was found to have the best MolDock score of −169.8 and form three hydrogen bonding interactions with Thr165 (3.20 Å), Asn46 (2.46 Å) and Gly77 (3.30 Å) (Figure 3). Figure 4 shows that compound **9f** was superimposed with co-crystalline clorobiocin in the active site of the *E. coli* 24 kDa domain (Figure 4). 

**Figure 5:**
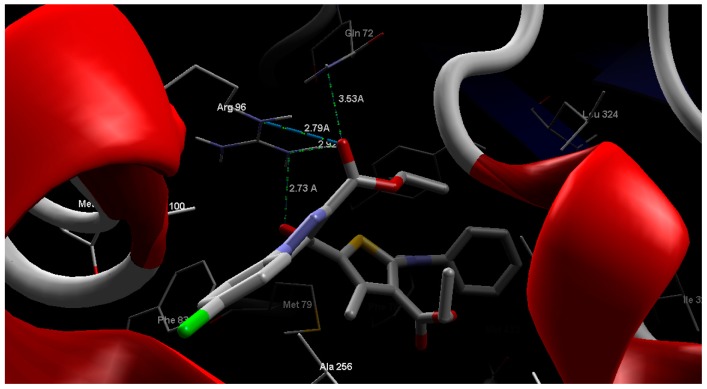
Interaction of compound **9b** with the active site of cytochrome P450 14α-sterol demethylase.

Regarding cytochrome P450 14α-sterol demethylase, the fourteen tested compounds revealed MolDock scores between −139.61 to −221.17 (Table 5). Compound **9b** was found to have best MolDock score of −221.17 and form four hydrogen bonding interactions with Arg96 (2.73, 2.79, 2.92 Å) and Gln72 (3.53 Å) (Figure 5). 

**Figure 6:**
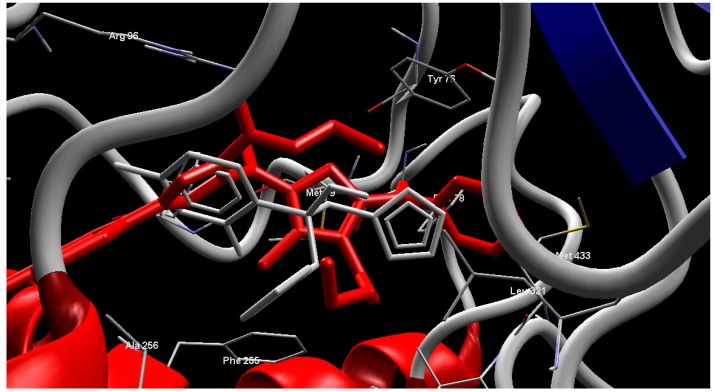
Superimpose of the co-crystallized fluconazole (**Gray**) and compound **9b** (**Red**) in the active site of cytochrome P450 14α-sterol demethylase.

Figure 6 shows the compound **9b** was superimposed with co-crystalline fluconazole in the active site of cytochrome P450 14α-sterol demethylase from *Mycobacterium tuberculosis* (*Mycobacterium* P450 DM).

## 3. Experimental Section

### 3.1. General

All melting points were recorded on a Gallenkamp melting point apparatus in open glass capillaries and are uncorrected. All the chemicals were purchased from Fluka Chemie GmbH (Buchs, Switzerland) and Sigma-Aldrich (Gillingham, Dorset, UK) and were used without further purification, unless otherwise stated. IR spectra were recorded as KBr pellets on a 6700 FT-IR Nicolet spectrophotometer (Thermo Fisher Scientific, Madison, WI, USA). Elemental analysis was carried out on a 2400 Elemental Analyzer, CHN mode (Perkin Elmer, Waltham, MA, USA). The NMR spectra were recorded on an Avance AV-600 NMR spectrometer (Bruker, International Equipment Trading Ltd 960 Woodlands Parkway, Vernon Hills, IL, USA). ^1^H-NMR (600 MHz) and ^13^C-NMR (150 MHz) were run in deuterated dimethyl sulphoxide (DMSO-*d*_6_) or deuterated dimethylformamide (DMF-*d*_7_). MS were recorded using a JMS-600 H instrument (Peabody, MA, USA). 

### 3.2. Preparation of Ethyl 5-Acetyl-4-methyl-2-(phenylamino)thiophene-3-carboxylate (**2**)

A mixture of ethyl acetoacetate (**1**, 13 g, 0.1 mol) and anhydrous potassium carbonate (25 g) in DMF (30 mL) was stirred vigorously at RT for 5 min then phenyl isothiocyanate (13.5 mL, 0.1 mol) was added with continued stirring for 30 min. The resulting reaction mixture was cooled in an ice bath, and chloroacetone (13.9 mL, 0.1 mol) was added over 15 min with continued stirring. The cooling bath was subsequently removed and the mixture was stirred for 2 h. The solid product **2** was precipitated by addition of H_2_O, collected by filtration, washed with water, and dried. Compound **2** was recrystallized from EtOH to afford bright yellow needles; Yield: 92%; m.p. 110 °C; IR (KBr, cm^−1^) ν_max_ = 3465, 1655, 1618, 1253 cm^−1^; ^1^H-NMR (600 MHz, DMSO-*d*_6_): δ 10.24 (brs, 1H, NH), 7.40–7.45 (m, 5 h, Ph), 4.32 (q, 2H, *J* = 6 Hz, CH_2_), 2.62 (s, 3H, CH_3_), 2.41 (s, 3H, CH_3_), 1.32 (t, 3H, *J* = 6 Hz, CH_3_); ^13^C-NMR (150 MHz, DMSO-*d*_6_): δ 14.68 (CH_3_CH_2_), 16.76 (CH_3_), 30.20 (CO-CH_3_), 60.96 (CH_3_CH_2_), 110.26, 121.31 (2C), 125.58, 130.27 (2C), 121.59, 140.36, 145.36, 162.81 (Ar-C), 165.86 (O-CO), 190.13 (CO-CH_3_); MS *m/z* (%): 303 [M^+^, 89%]; anal. calcd. for C_16_H_17_NO_3_S: C, 63.34; H, 5.65; N, 4.62; O, 15.82; S, 10.57; found: C, 63.35; H, 5.65; N, 4.63; S, 10.58. 

### 3.3. General Preparation of Compounds **3a**–**c** (GP1)

Compound **2** (0.303 g, 1 mmol) was fused with the appropriate aniline derivative (1 mmol, 1 equiv.) for 10 min, then EtOH (10 mL) was added to the reaction mixture, followed by ZnCl_2_ (0.2 gm) and the reaction mixture was refluxed for 6 h. The solid product was collected by filtration and recrystallized from EtOH to afforded **3a**–**c**.

*4-Methyl-2-phenylamino-5-(1-phenyliminoethyl)-thiophene-3-carboxylic acid ethyl ester* (**3a**). Compound **3a** was prepared as a beige powder in 99% yield according to GP1 using aniline (0.093 mL); m.p. 108–110 °C; IR (KBr, cm^−1^) ν_max_ = 3452, 1656, 1617 cm^−1^; ^1^H-NMR (600 MHz, DMSO-*d*_6_): δ 1.32 (t, 3H, *J* = 6 Hz, CH_2_CH_3_), 2.39, 2.65 (s, 3H, CH_3_), 4.30 (q, 2H, *J* = 6 Hz, CH_2_CH_3_), 6.46–7.48 (m, 10H, Ar-H) , 10.23 (s, 1H, NH); ^13^C-NMR (150 MHz, DMSO-*d*_6_): δ 15.60 (CH_3_) , 29.47 (N=C-CH_3_), 13.50, 59.18 (Et-carbons), 109.08, 113.24 (2C), 115.03, 120.14, 120.42 (2C), 124.43, 128.18 (2C), 129.11 (2C), 139.17, 144.23, 161.67 (Ar-C), 164.71 (C=N), 189.01 (C=O); MS *m/z* (%): 378.14 [M^+^, 65%]; Anal. calcd. for C_22_H_22_N_2_O_2_S: C, 69.81; H, 5.86; N, 7.40; S, 8.47; found: C, 69.82; H, 5.85; N, 7.42; S, 8.48.

*5-[1-(4-Chlorophenylimino)-ethyl]-4-methyl-2-phenylaminothiophene-3-carboxylic acid ethyl ester* (**3b**). Compound **3b** was prepared as a yellowish white powder in 95% yield according to GP1 using *p*-chloroaniline (0.127 g); m.p. 168–171 °C; IR (KBr, cm^−1^) ν_max_ = 3464, 1665, 1618 cm^−1^; ^1^H-NMR (600 MHz, DMSO-*d*_6_): δ 1.42 (t, 3H, *J* = 6 Hz , CH_2_CH_3_), 2.47, 2.75 (s, 3H, CH_3_), 4.37 (q, 2H, *J* = 6 Hz, CH_2_CH_3_), 6.60–7.10 (m, 4H, Ar-H ), 7.15–7.43 (m, 5H, Ar-H ),10.62 (s, 1H, NH); ^13^C-NMR (150 MHz, DMSO-*d*_6_): δ 16.77 (CH_3_), 30.32 (N=C-CH_3_), 14.33, 60.65 (Et-carbons), 109.69, 116.30 (2C), 120.29 (2C),120.65, 124.71, 129.12 (2C), 129.67 (2C), 139.72, 145.95 (Ar-C), 162.92 (C=N), 167.07 (C=O); MS *m/z* (%): 412.10 [M^+^, 10%]; Anal. calcd. for C_22_H_21_ClN_2_O_2_S: C, 63.99; H, 5.13; Cl, 8.59; N, 6.78; S, 7.77; found: C, 63.98; H, 5.14; Cl, 8.60; N, 6.80; S, 7.76.

*5-[1-(4-Methoxyphenylimino)-ethyl]-4-methyl-2-phenylaminothiophene-3-carboxylic acid ethyl ester* (**3c**). Compound **3c** was prepared as a pale yellow powder in 99% yield according to GP1 using *p*-methoxyaniline (0.123 gm); m.p. 155–157 °C; IR (KBr, cm^−1^) ν_max_ = 3451, 1656, 1619 cm^−1^; ^1^H-NMR (600 MHz, DMF*-d*_7_): δ 1.39 (t, 3H, *J* =8 Hz, CH_2_CH_3_), 2.21, 2.47 (s, 3H, CH_3_), 3.67 (s, 3H, OCH_3_), 4.39 (q, 2H, *J* = 8 Hz, CH_2_CH_3_), 6.63–7.52 (m, 9H, Ar-H), 10.45 (s, 1H, NH); ^13^C-NMR (150 MHz, DMF*-d*_7_): δ 16.16 (CH_3_), 55.25 (O-CH_3_), 13.94, 60.70 (Et-carbons), 110.05, 114.61 (2C), 115.45 (2C), 121.07 (2C), 121.39, 125.13, 129.93 (2C), 140.30, 143.01, 145.109 (Ar-C), 166.15 (C=N), 189.72 (C=O); MS *m/z* (%): 408.15 [M^+^, 89%]; Anal. calcd. for C_23_H_24_N_2_O_3_S: C, 67.62; H, 5.92; N, 6.86; S, 7.85; found: C, 67.63; H, 5.91; N, 6.84; S, 7.84.

### 3.4. Preparation of (E)-Ethyl 5-(3-(dimethylamino)acryloyl)-4-methyl-2-(phenylamino)thiophene-3-carboxylate (**4**)

A mixture of ethyl 5-acetyl-4-methyl-2-(phenylamino)thiophene-3-carboxylate (**2**, 5 mmol), and DMF-DMA (1.19 mL, 0.01 mol) was refluxed in *m*-xylene (15 mL) for 2 h. After cooling, the resulting solid product was collected by filtration and recrystallized from pet. ether to give the desired product **4** as a yellow powder; Yield: 93%; m.p. 118–120 °C; IR (KBr, cm^−1^) ν_max_ = 3465, 1705, 1637 cm^−1^; ^1^H-NMR (600 MHz, DMSO-*d*_6_): δ 1.27 (t, 3H, *J* = 6 Hz, CH_2_CH_3_), 2.45 (s, 3H, CH_3_), 2.78 (s, 3H, CH_3_), 3.05 (s, 3H, CH_3_), 5.32 (t, 1H, *J* = 12 Hz, CH), 7.58 (t, 1H, *J* = 12 Hz, CH), 7.13–7.45 (m, 10H, Ar-H), 10.15 (s, 1H, NH; ^13^C-NMR (150 MHz, DMSO-*d*_6_): δ 14.15 (CH_3_CH_2_), 16.49 (CH_3_), 37.19, 43.22 (N-CH_3_), 60.12 (CH_3_-CH_2_), 93.81 (-CO-CH=), 109.41, 119.92 (2C), 123.11, 124.03, 129.65 (2C), 139.05, 140.18, 159.48 (Ar-C), 153.08 (=CH-N), 165.62 (C=O) for ester, 180.01 (C=O) for enaminone; MS *m/z* (%): 385 [M^+^, 33%]; Anal. calcd. for C_19_H_22_N_2_O_3_S: C, 63.66; H, 6.19; N, 7.82; S, 8.95; found: C, 63.67; H, 6.20; N, 7.85; S, 8.97.

### 3.5. General Preparation of compounds **6a**–**c** (GP2)

A mixture of **4** (1 mmol) and the appropriate hydrazine derivative (1 mmol, 1 equiv.) was refluxed for 6 h in EtOH (10 mL). The solid product was collected by filtration and recrystallized from EtOH to afforded **6a**–**c**.

*Ethyl 4-methyl-2-(phenylamino)-5-(1H-pyrazol-3-yl)thiophene-3-carboxylate* (**6a**). Compound **6a** was prepared as a pale brown powder in 90% yield according to GP2 using hydrazine (1 mmol); m.p. 126–128 °C; IR (KBr, cm^−1^) ν_max_ = 3423, 3242, 1636, 1242 cm^−1^; ^1^H-NMR (600 MHz, DMSO-*d*_6_): δ 1.30 (t, 3H, *J* = 6 Hz, CH_2_CH_3_), 2.45 (s, 3H, CH_3_), 4.28 (q, 2H, *J* = 6 Hz, CH_2_CH_3_), 6.43 (s, 1H, CH), 7.76 (s, 1H, CH) ,7.08–7.39 (m, 5H, Ar-H), 10.01 (s, 1H, NH), 12.91 (s, 1H, NH); ^13^C-NMR (150 MHz, DMSO-*d*_6_): δ 13.96 (CH_2_CH_3_), 15.66 (CH_3_), 59.60 (CH_2_CH_3_), 103.81, 107.89, 111.55, 118.35, 122.72, 129.08, 130.47, 132.03, 140.15, 143.09, 157.96 (Ar-C), 166.17 (C=O); MS *m/z* (%): 327 [M^+^, 15%]; Anal. calcd. for C_17_H_17_N_3_O_2_S: C, 62.36; H, 5.23; N, 12.83; S, 9.79; found: C, 62.35; H, 5.23; N, 12.80; S, 9.76.

*Ethyl 4-methyl-5-(1-phenyl-1H-pyrazol-3-yl)-2-(phenylamino)thiophene-3-carboxylate* (**6b**). Compound **6b** was prepared as a reddish brown powder in 30% yield according to GP2 using aniline (0.093 mL); m.p. 166 °C; IR (KBr, cm^−1^) ν_max_ = 3445, 1655, 1597 cm^−1^; ^1^H-NMR (600 MHz, DMSO-*d*_6_): δ 1.33 (t, 3H, *J =* 6 Hz, CH_2_CH_3_), 2.77 (s, 3H, CH_3_), 4.35 (q, 2H, *J* = 6 Hz, CH_2_CH_3_), 7.04 (s, 1H, ^4^CH), 6.60–7.60 (m,10H,Ar-H), 8.33 (s, 1H, ^5^CH), 10.97 (s, 1H, NH); ^13^C-NMR (150 MHz, DMSO-*d*_6_): δ 14.75 (CH_2_CH_3_), 17.75 (CH_3_), 60.13 (CH_2_CH_3_), 112.77, 113.21, 115.41, 120.87, 122.14, 125, 125.65, 129.33, 130.22, 130.27, 140.05, 144.10, 147.59, 149.69 (Ar-C), 165.99 (C=O). MS *m/z* (%): 403 [M^+^, 10%]; Anal. calcd. for C_22_H_21_ClN_2_O_2_S: C, 68.46; H, 5.25; N, 10.41; S, 7.95; found: C, 68.47; H, 5.26; N, 10.40; S, 7.96.

*Ethyl 5-(1-(4-chlorophenyl)-1H-pyrazol-3-yl)-4-methyl-2-(phenylamino)thiophene-3-carboxylate* (**6c**). Compound **6c** was prepared as a reddish brown powder in 99% yield according to GP2 using *p*-chloroaniline (0.127 g); m.p. 163 °C; IR (KBr, cm^−1^) ν_max_ = 3427, 1653, 1593 cm^−1^; ^1^H-NMR (600 MHz, DMSO-*d*_6_): δ 1.26 (t, 3H, *J =* 6 Hz, CH_2_CH_3_), 1.90 (s, 3H, CH_3_), 4.24 (q, 2H, *J* = 6 Hz, CH_2_CH_3_), 6.62 (s, 1H, ^4^CH), 6.70–7.70 (m, 9H, Ar-H), 7.81 (s, 1H, ^5^CH), 10 (s, 1H, NH); ^13^C-NMR (150 MHz, DMSO-*d*_6_): δ 14.66 (CH_2_CH_3_), 16.33 (CH_3_), 60.63 (CH_2_CH_3_), 106.59, 108.17, 108.33, 111.64, 119.88 (2C), 120.29 (2C), 124.34, 126.02, 129.74 (2C), 130.18 (2C), 132.40, 134.77, 136.40, 141.20, 160.13 (Ar-C), 165.89 (C=O); MS *m/z* (%): 437 [M^+^, 89%]; Anal. calcd. for C_23_H_20_ClN_3_O_2_S: C, 63.08; H, 4.60; Cl, 8.10; N, 9.59; S, 7.32; found: C, 63.10; H, 4.60; Cl, 8.13; N, 9.562; S, 7.34.

### 3.6. General Preparation of Compounds **9a**–**f** (GP3)

To a mixture of the appropriate (*Z*)-ethyl 2-chloro-2-(2-phenylhydrazono)acetate derivative (1 mmol) in dry benzene (20 mL) containing NEt_3_ (a few drops) the enaminone **4** (1 mmol) was added, followed by ZnCl_2_ (0.2 gm) and the reaction mixture was then refluxed for 6 h. The solid product was collected by filtration and recrystallized from EtOH to afforded **9a**–**f**.

*Ethyl 4-(4-(ethoxycarbonyl)-3-methyl-5-(phenylamino)thiophene-2-carbonyl)-1-phenyl-1H-pyrazole-3-carboxylate* (**9a**). Compound **9a** was prepared as a yellowish white powder in 63% yield according to GP3 using (*Z*)-ethyl 2-chloro-2-(2-phenylhydrazono)acetate; m.p. 143–145 °C; IR (KBr, cm^−1^) ν_max_ = 3447, 1721, 1657, 1598 cm^−1^; ^1^H-NMR (600 MHz, CDCl_3_): δ 1.23, 1.40 (t, 3H, *J* = 6 Hz, CH_2_CH_3_), 2.63 (s, 3H, CH_3_), 4.31, 4.36 (q, 2H, *J* = 6 Hz, CH_2_CH_3_), 7.13–7.77 (m, 10H, Ar-H), 8.12 (s, 1H, pyrazol-H), 10.73 (s, 1H, NH); ^13^C-NMR (150 MHz, DMSO-*d*_6_): δ 12.88, 13.21 (CH_2_CH_3_), 15.95 (CH_3_), 59.67 (CH_2_CH_3_), 119.05, 123.89, 127.55, 128.02, 128.62, 137.22, 138.05, 142.05, 147.86, 148.01, 163.50; MS *m/z* (%): 503 [M^+^, 67%]; Anal. calcd. for C_27_H_25_N_3_O_5_S: C, 64.40; H, 5.00; N, 8.34; S, 6.37; found: C, 64.42; H, 5.01; N, 8.35; S, 6.39.

*Ethyl 1-(4-chlorophenyl)-4-(4-(ethoxycarbonyl)-3-methyl-5-(phenylamino)thiophene-2-carbonyl)-1H-pyrazole-3-carboxylate* (**9b**). Compound **9b** was prepared as yellowish white powder in 65% yield according to GP3 using (*Z*)-ethyl 2-chloro-2-(2-(4-chlorophenyl)hydrazono)acetate; m.p. 205–209 °C; IR (KBr, cm^−1^) ν_max_ = 3449, 1723, 1655, 1507 cm^−1^; ^1^H-NMR (600 MHz, DMSO-*d*_6_): δ 1.23 (t, 3H, *J* = 6 Hz, CH_2_CH_3_), 1.40 (t, 3H, *J* = 6 Hz, CH_2_CH_3_) 2.64 (s, 3H, CH_3_), 4.31 (q, 2H, *J* = 6 Hz, CH_2_CH_3_), 4.37 (q, 2H, *J* = 6 Hz, CH_2_CH_3_), 7.13–7.39 (m, 5H, Ar-H), 7.45–7.73 (m, 4H, Ar-H), 10.73 (s, 1H, NH); ^13^C-NMR (150 MHz, DMSO-*d*_6_): δ 12.93, 13.27 (CH_2_CH_3_), 16.02 (CH_3_), 59.76, 60.60 (CH_2_CH_3_), 109.35, 119.11, 119.91, 120.23, 123.81, 126.39, 127.42, 128.70, 128.75, 132.89, 136.44, 138.39, 141.85, 146.73, 160.08 (Ar-C), 162.81, 166.03 (C=O) ester, 179.73 (C=O); MS *m/z* (%): 537 [M^+^, 91%]; Anal. calcd. for C_27_H_24_ClN_3_O_5_S: C, 60.28; H, 4.50; Cl, 6.59; N, 7.81; S, 5.96; found: C, 60.27; H, 4.50; Cl, 6.62; N, 7.83; S, 5.95.

*Ethyl 4-(4-(ethoxycarbonyl)-3-methyl-5-(phenylamino)thiophene-2-carbonyl)-1-(p-tolyl)-1H-pyrazole-3-carboxylate* (**9c**). Compoun **9c** was prepared as a yellowish white powder in 50% yield according to GP3 using (*Z*)-ethyl-2-chloro-2-(2-(*p*-tolyl)-hydrazono)acetate; m.p. 170–172 °C; IR (KBr, cm^−1^) ν_max_ = 3428, 1721, 1656, 1618 cm^−1^; ^1^H-NMR (600 MHz, DMSO-*d*_6_): δ 1.12, 1.30 (t, 3H, *J* = 6 Hz, CH_2_CH_3_), 2.35 (s, 3H, CH_3_-Ph), 2.49 (s, 3H, CH_3_), 4.19, 4.31 (q, 2H, *J* = 6 Hz, CH_2_CH_3_), 7.19–7.79 (m,9H,Ar-H), 8.93 (s, 1H, pyrazole-H), 10.27 (s, 1H, NH).; ^13^C-NMR (150 MHz, DMSO-*d*_6_): δ 13.21, 13.45 (CH_2_CH_3_), 15.63, 15.88 (CH_3_), 59.73, 60.16 (CH_2_CH_3_), 109.35, 118.52, 120.57, 125.46, 129, 129.37 (Ar-C); MS *m/z* (%): 517 [M^+^, 95%]; Anal. calcd. for C_28_H_27_N_3_O_5_S: C, 64.97; H, 5.26; N, 8.12; S, 6.19; found: C, 64.97; H, 5.25; N, 8.15; S, 6.17.

*Ethyl 5-(3-acetyl-1-phenyl-1H-pyrazole-4-carbonyl)-4-methyl-2-(phenylamino)thiophene-3-carboxylate* (**9d**). Compound **9d** was prepared as a yellow powder in 67% yield according to GP3 using (*Z*)-2-oxo-Nʹ-phenylpropanehydrazonoyl chloride; m.p. 200–202 °C; IR (KBr, cm^−1^) ν_max_ = 3451, 1691, 1658, 1596 cm^−1^; ^1^H-NMR (600 MHz, CDCl_3_): δ 1.40 (t, 3H, *J* = 6 Hz, CH_2_CH_3_), 2.22 (s, 3H, CH_3_-CO), 2.63 (s, 3H, CH_3_), 4.36 (q, 2H, *J* = 6 Hz, CH_2_CH_3_), 7–7.76 (m, 9H, Ar-H) , 8.10 (s, 1H, pyrazole-H), 10.70 (s, 1H, NH); ^13^C-NMR (150 MHz, DMSO-*d*_6_): δ 14.32 (CH_2_CH_3_), 17.08 (CH_3_), 25.15 (CH_3_-CO), 60.67 (CH_2_CH_3_), 110.46, 119.81, 120.27, 120.37, 122.47, 125.96, 128.12, 128.50, 128.92, 129.76, 139.09, 139.60, 141.87, 147.58, 163.91 (Ar-C), 167, 181.48, 193.10 (C=O); MS *m/z* (%): 473 [M^+^, 75%]; Anal. calcd. for C_26_H_23_N_3_O_4_S: C, 65.94; H, 4.90; N, 8.87; O, 13.51; S, 6.77; found: C, 65.93; H, 4.90; N, 8.88; S, 6.78.

*Ethyl 5-(3-acetyl-1-(4-chlorophenyl)-1H-pyrazole-4-carbonyl)-4-methyl-2-(phenylamino)-thiophene-3-carboxylate* (**9e**). Compound **9e** was prepared as a yellow powder in 77% yield according to GP3 using (*Z*)-Nʹ-(4-chlorophenyl)-2-oxopropylhydrazonoyl chloride; m.p. 255–256 °C; IR (KBr, cm^−1^) ν_max_ = 3450, 1691, 1656, 1594 cm^−1^; ^1^H-NMR (600 MHz, DMSO-*d*_6_): δ 1.18 (t, 3H, *J* = 6 Hz, CH_2_CH_3_), 2.10 (s, 3H, CH_3_-CO), 2.49 (s, 3H, CH_3_), 4.28 (q, 2H, *J* = 6 Hz, CH_2_CH_3_), 7.13–8 (m, 9H, Ar-H), 8.96 (s, 1H, pyrazole-H), 10.28 (s, 1H, NH); ^13^C-NMR (150 MHz, DMSO-*d*_6_): δ 13.34 (CH_2_CH_3_), 15.69 (CH_3_), 24.59 (CH_3_-CO), 108.66, 115.65, 119.17, 120.54, 123.31, 128 .05, 128.39, 128.88, 129.01, 139.45; MS *m/z* (%): 507 [M^+^, 19%]; Anal. calcd. for C_26_H_22_ClN_3_O_4_S: C, 61.47; H, 4.37; Cl, 6.98; N, 8.27; S, 6.31; found: C, 61.48; H, 4.36; Cl, 7.00; N, 8.32; S, 6.30.

*Ethyl 5-(3-acetyl-1-(p-tolyl)-1H-pyrazole-4-carbonyl)-4-methyl-2-(phenylamino)thiophene-3-carboxylate* (**9f**). Compound **9f** was prepared as a yellow powder in 42% yield according to GP2 using (*Z*)-2-oxo-Nʹ-(*p*-tolyl)propanehydrazonoyl chloride; m.p. 215–217 °C; IR (KBr, cm^−1^) ν_max_ = 3451, 1658, 1634, 1595 cm^−1^; ^1^H-NMR (600 MHz, DMSO-*d*_6_): δ 1.32 (t, 3H, *J* = 6 Hz, CH_2_CH_3_), 2.10 (s, 3H, CH_3_-Ph), 2.49 (s, 3H, CH_3_), 2.72 (s, 3H, CH_3_-CO), 4.33 (q, 2H, *J* = 6 Hz, CH_2_CH_3_), 7.11–7.84 (m, 9H, Ar-H), 8.89 (s, 1H, pyrazol-H), 10.27 (s, 1H, NH); ^13^C-NMR (150 MHz, DMSO-*d*_6_): δ 14.32 (CH_2_CH_3_), 17.08 (CH_3_), 25.15 (CH_3_-CO), 60.67 (CH_2_CH_3_), 110.46, 119.81, 120.27, 120.37, 122.47, 125.96, 128.12, 128.50, 128.92, 129.76, 139.09, 139.60, 141.87, 147.58, 163.91 (Ar-C), 167, 181.48, 193.10 (C=O); MS *m/z* (%): 487 [M^+^, 55%]; Anal. calcd. for C_27_H_25_N_3_O_4_S: C, 66.51; H, 5.17; N, 8.62; S, 6.58; found: C, 66.50; H, 5.18; N, 8.61; S, 6.57.

### 3.7. Antifungal Activity of Compounds **2**, **3a**–**c**, **4**, **6a**–**c** and **9a**–**f**

Samples of **2**, **3a**–**c**, **4**, **6a**–**c** and **9a**–**f** were screened *in vitro* for antifungal activity against various fungi, namely, *Aspergillus fumigates*, *Syncephalastrum racemosum*, *Geotricum candidum* and *Candida albicans*. The antifungal activity was determined by the agar well diffusion method according to a reported procedure [27].

### 3.8. Antibacterial Activity of Compounds **2**, **3a**–**c**, **4**, **6a**–**c** and **9a**–**f**

Samples of **2**, **3a**–**c**, **4**, **6a**–**c** and **9a**–**f** were screened *in vitro* for antibacterial activity against various bacterial strains namely, *Escherichia coli and Pseudomoas aeruginosa* (Gram-negative bacteria) and *Bacillis subtilis and Staphylococcus pneumonia* (Gram-positive bacteria). The antibacterial activity was measured by the agar well diffusion method according to a reported procedure [27].

### 3.9. Molecular Modeling

For the docking of ligands into the proteins’ active sites and for estimating the binding affinities of docked compounds, the X-ray crystal structure of the *E. coli* 24 kDa domain in complex with clorobiocin (PDB code: 1KZN) and the crystal structure of cytochrome P450 14α-sterol demethylase (Cyp51) from *Mycobacterium tuberculosis* in complex with fluconazole (PDB 1EA1) were obtained from the Brookhaven Protein Data Bank [28] and loaded to the Molegro Virtual Docker (MVD2013.6.0.0 [win32]) program (fully functional free trial version with time limiting license [29]). The non-bonded oxygen atoms of water, present in the crystal structure, were removed. ChemBio3D Ultra 10 [30] was used to draw the 3D structures of the different ligands. Ligands were further pre-optimized using the free version of MarvinSketch 4.1.13 from Chemaxon Ltd. [31] with the MM force field and saved in Tripos mol2 file format. MolDock score functions were used with a 0.3 Å grid resolution. The binding sites were defined to any residues with 10 Å distant from the cocrystallized clorobiocin and fluconazole in the complex crystal structure of the enzymes [32,33].

## 4. Conclusions

The synthesis in excellent yield and characterization of the new compound ethyl 5-acetyl-4-methyl-2-(phenylamino)thiophene-3-carboxylate (**2**) was reported. The structure of **2** was deduced by single-crystal X-ray diffraction. New enaminone derivatives **4** and a series of novel pyrazole derivatives **6a**–**c** and **9a**–**f** were reported. All synthesized products have been examined for anti-microbial activity and shown promising results. Also the molecular docking of the synthesized compounds was discussed.

## References

[1] Ragavendran J.V., Sriram D., Patil S., Reddy I.V., Bharathwajan N., Stables J., Yogeeswari P. (2007). Design and synthesis of anticonvulsants from a combined phthalimide–GABA–anilide and hydrazone pharmacophore. Eur. J. Med. Chem..

[2] Dimmock J.R., Puthucode R.N., Smith J.M., Hetherington M., Wilson Quail J., Pugazhenthi U., Lechler T., Stables J.P. (1996). Anticonvulsant activities of some arylsemicarbazones displaying potent oral activity in the maximal electroshock screen in rats accompanied by high protection indices. J. Med. Chem..

[3] Polivka Z., Holubek J., Svatek E., Metys J., Protiva M. (1984). Regio-and stereo-selection reaction of 1,3-dialkyl-substituted allyl anions with aldehydes via *η*^3^-allyltitanium compounds. J. Chem. Soc. Chem. Commun..

[4] Shank R.P., Doose D.R., Streeter A.J., Bialer M. (2005). Plasma and whole blood pharmacokinetics of topiramate: The role of carbonic anhydrase. Epilepsy Res..

[5] Yogeeswari P., Thirumurugan R., Kavya R., Samuel J.S., Stables J.D. (2004). Sriram, 3-Chloro-2-methylphenyl-substituted semicarbazones: Synthesis and anticonvulsant activity. Eur. J. Med. Chem..

[6] Kucukguzel S.G., Mazi A., Sahin F., Ozturk S., Stables J. (2003). Synthesis and biological activities of diflunisal hydrazide-hydrazones. Eur. J. Med. Chem..

[7] Thirumurugan R., Sriram D., Saxena A., Stables J., Yogeeswari P. (2006). 2,4-Dimethoxyphenylsemicarbazones with anticonvulsant activity against three animal models of seizures: Synthesis and pharmacological evaluation. Bioorg. Med. Chem..

[8] Riaz N., Anis I., Aziz-ur-Rehman, Malik A., Ahmed Z., Muhammad P., Shujaat S., Atta-ur-Rahman (2003). Emodinol, *β*-Glucuronidase, inhibiting triterpine from Paeonia emodi. Nat. Prod. Res..

[9] Ahmad V.U., Khan A., Farooq U., Kousar F., Khan S.S., Nawaz S.A., Abbasi M.A., Choudhary M.I. (2005). Three new cholinesterase-inhibiting *cis*-clerodane diterpenoids from otostegia limbata. Chem. Pharm. Bull..

[10] Neto B.A.D., Lapis A.A.M., Bernd A.B., Russowsky D. (2009). Studies on the Eschenmoser coupling reaction and insights on its mechanism. Application in the synthesis of Norallosedamine and other alkaloids. Tetrahedron.

[11] Cvetovich R.J., Pipik B., Hartner F.W., Grabowski E.J. (2003). Rapid synthesis of tetrahydro-4*H*-pyrazolo[1,5-*a*]diazepine-2-carboxylate. Tetrahedron Lett..

[12] Zhang Z.H., Zhang X.N., Mo L.P., Li Y.X., Ma F.P. (2012). Catalyst-free synthesis of quinazoline derivatives using low melting sugar-urea-salt mixture as a solvent. Green. Chem..

[13] Siddiqui Z.N., Farooq F. (2012). Heterocyclic Silica supported sodium hydrogen sulfate (NaHSO_4_–SiO_2_): A novel, green catalyst for synthesis of pyrazole and pyranyl pyridine derivatives under solvent-free condition via heterocyclic *β*-enaminones. J. Mol. Catal. A Chem..

[14] Siddiqui Z.N., Ahmed N., Farooq F., Khan K. (2013). Highly efficient solvent-free synthesis of novel pyranyl pyridine derivatives via *β*-enaminones using ZnO nanoparticles. Tetrahedron Lett..

[15] Li M.Y., Xu H.W., Fan W., Ye Q., Wang X., Jiang B., Wan S.L., Tu S.J. (2014). New formal (3+3) cycloaddition of enaminones for forming tetracyclic indolo[2,3-*b*]quinolines under microwave irradiation. Tetrahedron.

[16] Abass M., Mostafa B.B. (2005). Synthesis and evaluation of molluscicidal and derived from 4-hydroxyquinolinones: Part IX. Bioorg. Med. Chem..

[17] Cox D.S., Scott K.R., Gao H., Eddington N.D. (2002). Effect of P-glycoprotein on the pharmacokinetics and tissue distribution of enaminone anticonvulsants: analysis by population and physiological approaches. J. Pharmacol. Exp. Ther..

[18] Cox D.S., Scott K.R., Gao H., Raje S., Eddington N.D. (2001). Influence of multidrug resistance (MDR) proteins at the blood-brain barrier on the transport and brain distribution of enaminone anticonvulsants. J. Pharm. Sci..

[19] Edafiogho I.O., Kombian S.B., Ananthalakshmi K.V., Salama N.N., Eddington N.D., Wilson T.L., Alexander M.S., Jackson P.L., Hanson C.D., Scott K.R. (2007). Enaminones Exploring additional therapeutic activities. Pharm. Sci..

[20] Mabkhot Y.N., Barakat A., Yousuf S., Choudhary M.I., Frey W., Ben Hadda T., Mubarak M.S. (2014). Substituted thieno[2,3-*b*]thiophenes and related congeners: Synthesis, *β*-glucuronidase inhibition activity, crystal structure, and POM analyses. Bioorg. Med. Chem..

[21] Mabkhot Y.N., Al-Majid A.M., Barakat A., Alshahrani S., Yamin S. (2011). 1,1'-(3-Methyl-4-phenylthieno[2,3-*b*]thiophene-2,5-diyl)diethanone as a building block in heterocyclic synthesis. Novel synthesis of some pyrazole and pyrimidine derivatives. Molecules.

[22] Mabkhot Y.N., Al-Majid A.M., Alamary A.S. (2011). Synthesis and chemical characterisation of some new diheteroaryl thienothiophene derivatives. Molecules.

[23] Mabkhot Y.N. (2009). Synthesis and analysis of some *bis*-heterocyclic compounds containing Sulphur. Molecules.

[24] Mabkhot N.Y., Barakat A., Al-Majid A.M., Choudhary M.I. (2013). Synthesis of thieno[2,3-*b*]thiophene containing *bis*-heterocycles-novel pharmacophores. Int. J. Mol. Sci..

[25] Sheldrick G.M. (2008). A short history of SHELX. Acta Crystallogr..

[26] Spek A.L. (2009). Structure validation in chemical crystallography. Acta Crystallogr..

[27] Smania A., Monache F.D., Smania E.F.A., Cuneo R.S. (1999). Antibacterial activity of steroidal compounds isolated from *Ganoderma applanatum* (Pers.) Pat. (Aphyllophoromycetideae) fruit body. Int. J. Med. Mushrooms.

[28] RCSB Protein Data Bank. http://www.rcsb.org/pdb.

[29] Molegro Virtual Docker (MVD 2013.6.0.0). http://www.molegro.com.

[30] Kerwin S.M. (2010). ChemBioOffice Ultra 2010 suite. J. Am. Chem. Soc..

[31] MarvinSketch, version 6.1.0. http://www.chemaxon.com/.

[32] Lafitte D., Lamour V., Tsvetkov P.O., Makarov A.A., Klich M., Deprez P., Moras D., Briand C., Gilli R. (2002). DNA gyrase interaction with coumarin-based inhibitors: The role of the hydroxybenzoate isopentenyl moiety and the 5'-methyl group of the noviose. Biochemistry.

[33] Banfi E., Scialino G., Zampieri D., Mamolo M.G., Vio L., Ferrone M., Fermeglia M., Paneni M.S., Pricl S. (2006). Antifungal and antimycobacterial activity of new imidazole and triazole derivatives. A combined experimental and computational approach. J. Antimicrob. Chemother..

